# Antibody-Drug Conjugates in Prostate Cancer: A Systematic Review

**DOI:** 10.7759/cureus.34490

**Published:** 2023-02-01

**Authors:** Mariana Sardinha, Ana Filipa Palma dos Reis, João Vasco Barreira, Mário Fontes Sousa, Simon Pacey, Ricardo Luz

**Affiliations:** 1 Medical Oncology, Centro Hospitalar Universitário Central Lisboa, Lisbon, PRT; 2 Medical Oncology, Addenbrooke's Hospital, Cambridge University Hospitals National Health Service Foundation Trust, Cambridge, GBR; 3 Medical Oncology, Hospital dos Serviços de Assistência Médico-Social, Lisbon, PRT; 4 Medical Oncology, Hospital Companhia União Fabril Tejo, Lisbon, PRT

**Keywords:** b7-h3, her 2, dll-3, tf, steap1, trop2, psma, prostate cancer, antibody drug conjugates

## Abstract

The prognosis in the setting of metastatic castration-resistant prostate cancer patients (mCRPC) remains limited. Therefore, novel treatment strategies remain an unmet need. Antibody-drug conjugates (ADC) emerged as a new drug concept with the potential to deliver a cytotoxic payload with limited off-target toxicity and potentially bystander effect. Following the success of ADCs in breast cancer and urothelial tumours, their activity in prostate cancer is now under investigation. Thus, the aim of this systematic review was to identify published and ongoing prospective clinical trials regarding ADC treatment in prostate cancer.

A systematic search of PubMed, MEDLINE, and Web of Science was conducted as per PRISMA guidelines to identify prospective clinical trials of ADCin prostate cancer. Trials are currently ongoing on ClinicalTrials.gov and in the EU. The Clinical Trials Register was also identified. Abstracts, publications in languages other than English, review articles, retrospective analyses, and phase I trials were excluded.

A total of six phase I/II prospective clinical trials already published were included. Seven ongoing trials were also identified. All studies were in the refractory/advanced tumour setting, and two included only mCRPC patients. The ADC targets were prostate-specific membrane antigen (PSMA), trophoblast cell surface antigen-2 (TROP-2), six-transmembrane epithelial antigen of prostate-1 (STEAP-1), tissue factor (TF), delta-like protein 3 (DLL-3), B7-H3 family of proteins (B7-H3), and human epidermal growth factor receptor 2 (HER2). Regarding the efficacy of PSMA ADC treatment in the second-line or beyond mCRPC setting, a PSA ≥ 50% decline rate in 14% of all treated patients was reported. One patient achieved a complete response with TROP-2 ADC. Overall, a wide range of safety issues were raised, particularly in connection with neuropathy and hematologic toxicity.

Novel therapies have been changing the scope of treatment in mCRPC. ADCs seem to provide efficacy benefits, even with potential toxicity. The results of most prospective ongoing studies are still awaited, and a longer follow-up time is warranted to evaluate the real impact of ADCs in PCa.

## Introduction and background

Prostate cancer (PCa) is the second most commonly diagnosed solid tumour and the fifth leading cause of cancer-related death in men worldwide, with 268,490 estimated new cases in the year 2022. Moreover, in the last decade, the incidence of metastatic PCa diagnosis has been rising, from 3.9% in 2007 to 8.2% in 2018 [1].

Albeit the improvements in diagnosis and treatment, a significant proportion of early-stage PCa patients will experience progression to metastatic castration-resistant prostate cancer (mCRPC) [2]. Furthermore, after progression on novel antiandrogen therapies and available standard chemotherapies, treatment sequencing and prognosis of refractory mCRPC remain a major challenge.

Thus, in the past two decades, the therapeutic scope of refractory mCRPC has been broadened, with new treatment approvals including radioisotope therapy, gene-directed therapy, and immune checkpoint inhibitors [2]. However, only a few patients are eligible for these drugs, and responses can be limited [3]. Therefore, the development of new agents and treatment strategies for mCRPC remains an important goal.

For this unmet need, recent advances in understanding the pathology of tumours led to the discovery of novel antigens overly expressed on cancer cells, that may be used as more specific therapeutic targets. In this setting, antibody-drug conjugates (ADCs) emerged as a new drug concept, using monoclonal antibodies (mAb) targeted to specific tumour antigens with a cytotoxic payload agent attached to the mAb. Therefore, enabling larger therapeutic windows and ultimately reducing off-target toxicities.

Furthermore, ADCs have shown great clinical activity in the treatment of other tumour types, especially in human epidermal growth factor receptor 2 (HER2)-positive breast cancer, with the success of ado-trastuzumab emtansine [4] and trastuzumab-deruxtecan (DXd) treatments [5].

**Figure 1:**
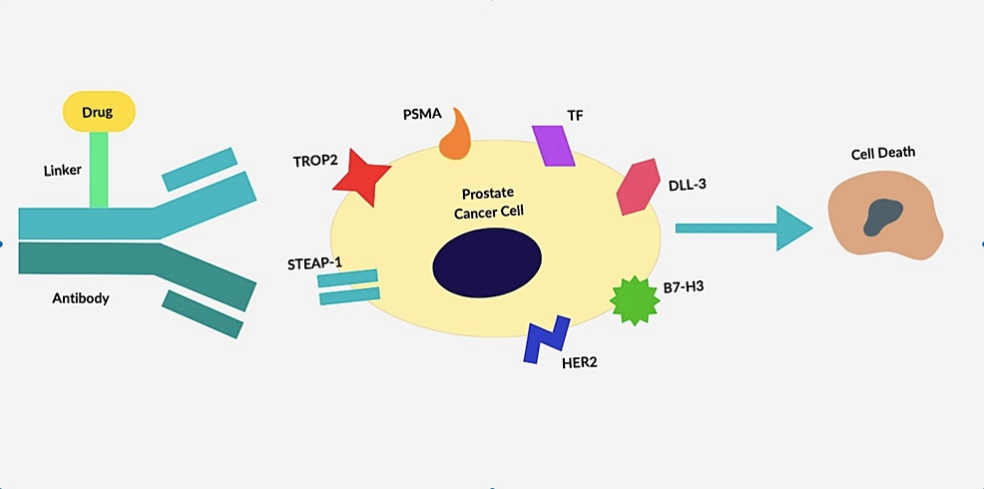
Antibody-drug conjugates and targets in prostate cancer cells. B7-H3: B7-H3 family of proteins; DLL-3: delta-like protein 3; HER2: human epidermal-growth factor receptor 2; PSMA: prostate-specific membrane antigen; STEAP-1: six-transmembrane epithelial antigen of prostate-1; TF: tissue factor; TROP-2: trophoblast cell surface antigen-2. Image credit: Mariana Sardinha.

ADCs have also been assessed in the uro-oncology field, with the approval of enfortumab-vedotin, an ADC for nectin cell adhesion molecule 4 (NECTIN-4), in patients with previously treated locally advanced or metastatic urothelial cancer [6]. As for prostate cancer, several antigens are currently under study as optimal therapeutic targets. ADCs and targets are represented in Figure 1.

This study aims to review the scope of efficacy evidence of ADCs in prostate cancer and the ongoing clinical trials as well.

## Review

Methods

Search Strategy

A systematic literature review was carried out in accordance with the Preferred Reporting Items for Systematic Reviews and Meta-Analyses (PRISMA) checklist [7]. The two authors performed an independent search in the PubMed, MEDLINE, and Web of Science databases to identify relevant studies regarding ADCs in prostate cancer.

A literature search was performed for the inclusion of English-language papers indexed to the PubMed, MEDLINE, and Web of Science databases in August 2022. To identify ongoing phase II and III prostate cancer trials, searches were performed on both the US National Library of Medicine database (ClinicalTrials.gov) and the European Union Clinical Trials Register (clinicaltrialsregister.eu).

The keywords and combinations used were: "antibody-drug conjugates" or "ADC" and "prostate cancer" and "PSMA" or "TROP2" or "STEAP1" or "TF" or "HER2" or "SLC44A4" or "B7-H3" or "CD46" or "TM4FS1" or "DLL-3," with no range date filter. The bibliography of each potentially eligible article was hand-searched for relevant studies. Trial registries of prospective studies were consulted at ClinicalTrials.gov and clinicaltrialsregister.eu.

In all three stages, data were extracted and analyzed by two authors independently. In the first phase, duplicate references were excluded. In the second stage, all titles and abstracts from unique references were screened. The authors selected which articles should have full-text revision by consensus. In the final stage, a full-text reading of all remaining references was performed. Peer review involved proofreading the syntax and overall structure.

Inclusion Criteria

**Figure 2:**
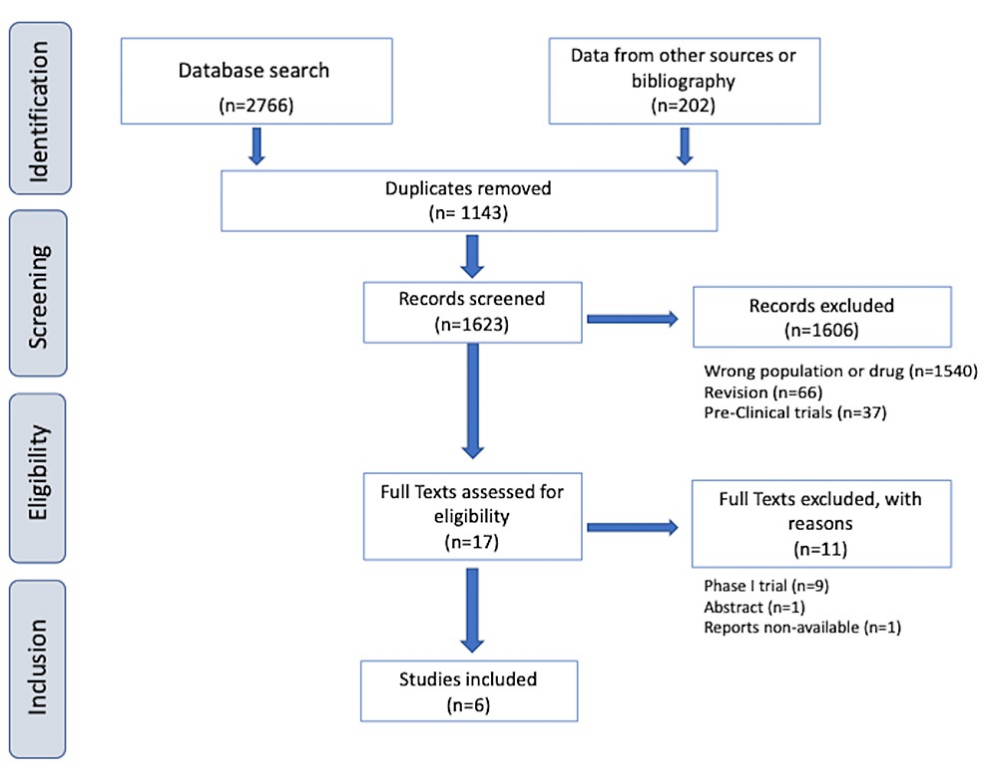
Inclusion and exclusion criteria for systematic review of phase I/II prospective clinical trials of ADCs in prostate cancer.

Full-text phase I/II published trials reporting outcomes on the use of ADCs for prostate cancer treatment were considered for inclusion, as summarized in Figure 2.

Exclusion Criteria

All abstracts, reviews, opinion-papers, retrospective analyses, pre-clinical studies and non-English language articles were excluded. Repeated publications on the same cohort were excluded as well. In order to narrow the scope of the review, phase I only trials were also excluded.

Data Extraction and Synthesis

All eligible studies with published results were analyzed, with the extraction of the following information: treatment intervention, number of enrolled patients, primary endpoint, objective response rate, as well as median progression-free and overall survival, when available.

The outcomes of the enrolled studies were compared but could not be combined due to their heterogeneity, small sample size, and different endpoints. The authors organized all eligible clinical trials by antibody target.

Results

From a total of 2766 records identified through the PubMed, MEDLINE, and Web of Science databases, 1143 duplicates were removed. In the second screening stage, 1606 records were excluded, leaving 17 full-text publications to analyze, of which 11 were excluded for not meeting the inclusion criteria (Figure 2). Six prospective phase I/II clinical trials focusing on prostate cancer treatment were included in this review and are summarized in Table 1.

**Table 1:** Results of phase I/II prospective studies of ADC in Prostate Cancer. ChT: chemoterapy; DLL-3: delta-like protein 3; mCPRPC: metastatic castration-resistant prostate cancer; MMAE: monomethyl auristatin E; ORR: overall response rate; OS: overall survival; PCa: prostate cancer; PSA: prostate-specific antigen; PSMA: prostate-specific membrane antigen; STEAP-1: six-transmembrane epithelial antigen of prostaZte-1; TF: tissue factor; Trop-2: trophoblast cell surface antigen-2.

Study/reference	Report date	Phase	Enrollment (n)	Antibody target	Intervention	Payload	Disease setting	Inclusion criteria	Histology	Primary endpoint	ORR (%)	Median OS
Petrylak et al. [8]	2020	II	119	PSMA	PSMA-MMAE	Monomethyl auristatin E	mCRPC < 2 prior lines of treatment	Disease progression after enzalutamide/abiraterone acetate	Prostate carcinoma	PSA response	14%	91.7%-ChT treated 97.1%-ChT naíve
Milowsky et al. [9]	2016	I/II	62	PSMA	MLN2704	Maytansinoid-1	mCRPC > 1 prior line of treatment	Disease progression on hormonotherapy and/or ChT	Prostate carcinoma	PSA response	8%	Not reported
Bradia et al. [10]	2021	I/II	11	Trop-2	Sacituzumab-Govitecan	SN-38	Relapsed or refractory PCa > 1 prior line of treatment	Refractory metastatic epithelial cancers	Prostate carcinoma	ORR	9.1%	Not reported
Carrasquillo et al. [11]	2019	II	19	STEAP-1	^89^Zr-DFO + MSTP2109A	Monomethyl auristatin E	mCRPC > 1 prior line of treatment	STEAP-1 antigen presence of >1+ in tumor tissue	Prostate carcinoma	Safety	Not reported	Not reported
de Bono et-al [12]	2019	I/II	12	TF	Tisotumab vedotin	Monomethyl auristatin E	Metastatic PCa > 3 prior lines of treatment	Relapsed, advanced, or metastatic cancer	Prostate carcinoma	ORR	0%	Not reported
Mansfield et al. [13]	2021	I/II	21	DLL-3	Rovalpituzumb tesirine	Pyrrolobenzodiazepine	Unresectable, refractory or advanced solid tumors	DLL-3 immunohistochemical staining in >1% of tumor cells	Neuroendocrine prostate tumors	Safety	7%	6.4 months (4-29)

Three trials exclusively enrolled prostate cancer patients, whereas the remainder also included patients with other solid tumors. One trial included neuroendocrine prostate cancer (NEPC) histology. All trials included only advanced prostate cancer that was refractory to prior treatments. Different primary endpoints were seen throughout the trials. An additional 22 trials were identified on clinicaltrials.gov, of which only seven were phase I/II. Six trials were actively enrolling patients, and one study was active but not recruiting. A similar search on the EU clinical trials registry yielded 90 results, of which three met the prespecified criteria. All three were duplicates, previously identified on clinicaltrials.gov. The phase I/II ongoing trials are listed in Table 2.

**Table 2:** Phase I/II ongoing prospective clinical trials of ADC in prostate cancer. B7-H3: B7-H3 family of proteins; Dato-Dxd: datopotamab-deruxtecan; mCPRPC: metastatic castration-resistant prostate cancer; HER2: human epidermal-growth factor receptor 2; PARPi: poly (ADP-ribose) polymerase inhibitors; TROP-2: trophoblast cell surface antigen-2; ORR: overall response rate.

Study/reference	Launch date	Phase	Antibody target	Intervention	Payload		Disease setting	Histology		Primary endpoint	Activity
NCT04381832 [14]	2020	I/II	TROP-2	Sacituzumab-Govitecan + adenosine receptor antagonist combinations		SN-38	mCRPC after progression on abiraterone and < 2 prior lines of chemotherapy	Prostate carcinoma	ORR		Active, in recruitment
NCT03725761 [15]	2018	I/II	TROP-2	IMMU-132		SN-38	mCRPC > 1 prior line of enzalutamide or abiraterone	Prostate carcinoma	PSA response rate		Active, in recruitment
NCT05489211 [16]	2022	I/II	TROP-2	Dato-DXd monotherapy and in combination		DXd	Advanced or metastatic solid tumors	Multiple	ORR		Active, in recruitment
NCT04644068 [17]	2020	I/II	HER2/TROP-2	Trastuzumab-DXd + PARPi/Dato-DXd + PARPi		DXd	Advanced or metastatic solid tumors	Multiple	Safety		Active, in recruitment
NCT02465060 [18]	2015	II	HER2	Ado-trastuzumab emtansine		Maytansinoid-1	Advanced refractory solid tumors/lymphomas/multiple myeloma	Multiple	ORR		Active, in recruitment
NCT03602079 [19]	2018	II	HER2	A166		Duostatin-5	Refractory locally advanced/metastatic solid tumors with HER2 expression or amplification	Multiple	Safety		Active, not recruiting
NCT03729596 [20]	2018	II	B7-H3	MGC018		Duocarmycin	Advanced solid tumors	Multiple	Safety		Active, in recruitment

This review identified five main molecular targets with existing ADC treatments: PSMA, TROP-2, STEAP1, TF, and DLL-3. Current ongoing trials involving alternative targets, such as HER2 and B7-H3, are also presented in the results.

PSMA

Prostate-specific membrane antigen (PSMA) is a membrane glycoprotein expressed primarily in prostatic tissue with higher-extent staining seen in the malignant prostatic epithelial cells. It is a well-established target in both the diagnostic and therapeutic fields of prostate cancer, with a total of four registered trials evaluating the efficacy of PSMA-directed ADC treatments. Two of them have published results, and the remaining two are completed but without results. In accordance with the exclusion criteria, the last two were not included in this study.

In one open-label, single-arm phase II trial [8], anti-PSMA ADC linked to monomethyl auristatin E (MMAE) was studied in 119 mCRPC patients, 84 of whom received prior chemotherapy, and the remaining 35 were chemotherapy-naïve. The primary endpoints were antitumour activity [best decline in prostate-specific antigen (PSA), circulating tumour cells (CTC) from baseline, and tumour radiologic response] and safety analyses in both groups of patients.

A total of 77 patients had CTC counts elevated at baseline (≥5 cells/7.5 mL blood) and at least one value after treatment. A rising PSA was not mandatory for entry into this study. After disease progression (40.3%), toxicity was the most common reason to discontinue treatment (31.3%). Two deaths from sepsis and neutropenia were reported to be directly related to the study drug. These dose-limiting events occurred in two chemotherapy-experienced patients during cycle 1, and after an initial safety review, the dose was reduced from 2.5 to 2.3 mg/kg.

In the overall population, 14% reported a PSA ≥50% decline rate. When compared to the group of previously treated patients, PSA and CTC declines of ≥50% were more frequent in the chemotherapy-naïve group of patients: 21% (n=7) versus 14% (n=12) and 89% (n=31) versus 78% (n=66), respectively.

Regarding the overall radiologic response according to response evaluation criteria in solid tumours (RECIST), a partial response was seen in 5.7% (n=2) of chemotherapy-naïve patients, and the stable disease rate was higher in the chemotherapy-naïve group (68.7% vs. 60.7%). In a seven-month follow-up period, a superior OS was seen in the chemotherapy-free group (97.1% vs. 91.7%).

Another open-label, single-arm phase I/II clinical trial [9] included 62 patients with progressive mCRPC who were treated with ascending doses of MLN2704, an anti-PSMA ADC linked to the antimicrotubule chemotherapeutic drug maytansinoid-1 (DM1). Primary endpoints were dose-limiting toxicity, maximum tolerated dose, and ≥50% decline from baseline PSA without evidence of disease progression.

Fifteen patients (38%) discontinued treatment due to adverse events (AE). Neurotoxicity was dose-limiting, with 44 patients (71%) exhibiting peripheral neuropathy and 6 (10%) classified as having a serious AE. These AE were considered dose-dependent. Regarding anti-tumour efficacy, 8% (n=5) presented a ≥50% decline in PSA for a median duration of 86 days (56-520 days), and 8% (n=5) had PSA stabilization lasting ≥90 days.

TROP-2

The second group is trophoblast cell surface antigen-2 (Trop-2), a transmembrane calcium signal transducer overly expressed in many epithelial cancers and believed to be correlated with disease progression and the development of metastases, including prostate cancer [10]. Four trials are currently active and enrolling prostate cancer patients.

Sacituzumab govitecan (SG), a Trop-2-directed antibody-drug conjugate with SN-38 as payload, has been investigated in the IMMU-132 phase I/II basket multicenter trial [10]. A total of 495 patients with various advanced epithelial cancers and progressive disease after at least one standard treatment regimen were included, regardless of TROP-2 expression. The primary endpoints were overall safety and efficacy data.

Toxicities were accountable for 51.7% of treatment interruptions and the most commonly reported treatment-related AE were neutropenia (21%) and anemia (5.3%). Dose delay/reduction and/or granulocyte colony-stimulating factor for neutropenia were used to manage treatment toxicity. Febrile neutropenia (4%) and diarrhoea (2.8%) were the most common serious AEs reported. One treatment-related death due to an aspiration pneumonia event was registered.

Eleven patients with metastatic castration-resistant prostate cancer participated. Regarding efficacy data, an ORR of 9.1% was reported, with one patient achieving a complete response. Stable disease was reported in 36.4% (n=4) of patients. Three active recruiting phase I/II trials are studying TROP-2 ADC and are listed in Table 2.

The first trial investigated Etrumadenant, an adenosine receptor antagonist, in combination with Sacituzumab-Govitecan in the mCRPC setting after progression on abiraterone and 2 or fewer prior lines of chemotherapy [14]. The second trial [16] is studying Datopotamab-Deruxtecan (Dato-DXd), a humanized monoclonal antibody against TROP-2 linked to the deoxyribonucleic acid (DNA) topoisomerase I inhibitor DXd. Dato-DXd is being studied alone or in combination with other drugs in advanced or metastatic solid tumours.

The third trial is investigating IMMU-132, a monoclonal antibody targeting TROP-2 and conjugated to the active metabolite of irinotecan (SN38), in the mCRPC setting after at least one prior line of enzalutamide or abiraterone [15].

STEAP1

The third group is the relatively new six-transmembrane epithelial antigen of prostate-1 (STEAP-1) family of proteins, localized in the plasma membrane of epithelial cells and known to have a role in cell adhesion and tumour invasiveness. STEAP1 is primarily expressed in prostate cells and is either low or absent in normal tissues. It is a potential cell surface target for imaging and therapeutic interventions in this setting.

STEAP-1 ADC was evaluated in an open-label, single-center, phase I/II trial [11], in which 19 patients with documented metastatic CRPC were prospectively imaged with positron emission tomography (PET/CT) with 89Zr-DFO-MSTP2109A, a radiolabeled antibody that recognizes STEAP-1. All patients had an immunohistochemistry of STEAP-1 antigen presence of 1+ or above on tumour tissue. The primary endpoint was to assess the ability to detect disease lesions and also assess the safety data.

Fifteen of these patients were enrolled in a parallel phase I therapeutic trial [21] with DSTP3086S, an ADC based on monomethyl auristatin E, a microtubule inhibitor conjugated to MSTP2109A, not included in this review. However, some information about the ADC activity in this group of patients was detailed in this phase I/II trial.

Reportedly, the patients received 1-21 cycles of DSTP3086S (a median of five cycles). Because of AE, six patients (40%) had to discontinue treatment. Only nine patients continued to be treated until progression. Overall, there were only minor responses to ADC treatment (data not shown). No correlations were found between the maximum standardized tumour uptake value and the PSA variation. No significant toxicity was reported in this trial.

Regarding tumour uptake on PET/CT with 89Zr-DFO-MSTP2109A and detection of soft tissue and bone disease, there was an estimate of 86% of histologically positive lesions being true positives (95% confidence interval, 75-100%).

TF

The fourth group is tissue factor (TF), a transmembrane glycoprotein that functions as the main initiator of the extrinsic coagulation pathway and that is also accountable for cell-signalling properties associated with poor clinical outcomes (tumour growth, angiogenesis, and metastasis).

InnovaTV 201 is a multicentric, open-label, phase I/II trial [12], which studied tisotumab vedotin, an anti-TF ADC conjugated with monomethyl auristatin E in one dose escalation phase, followed by a dose expansion phase. A total of 27 patients with relapsed, advanced, or metastatic solid tumours were enrolled in the dose-escalation phase, four of them with prostate cancer. Expression of tissue factor was not needed. Afterwards, 147 patients were treated with the recommended phase 2 dose in the dose expansion phase, 8 of whom were prostate cancer patients. The primary endpoint was the safety and tolerability of all patients who received at least one dose of tisotumab vedotin. Anti-tumour activity was a secondary endpoint.

Serious AE related to the study drug was reported in 27% of patients (n=39), more frequently associated with vomiting, abdominal pain, and anaemia. Nine deaths were reported during the study: three in the dose escalation phase and six in the dose expansion phase. Only one death, pneumonia in the dose expansion phase, was directly related to the study drug.

In the dose-expansion phase, the median follow-up time at data cutoff was 2.8 months, with an ORR of 12.6% in the entire treated population. Neither radiographic imaging nor prostate-specific antigen concentration responses were reported in prostate cancer patients.

DLL-3

The last group is delta-like protein 3 (DLL-3), a ligand in the Notch signalling pathway that is highly expressed in tumours of neuroendocrine origin, such as neuroendocrine carcinomas (NEC) or neuroendocrine tumours (NET), which are related to cell cycle development and death signaling.

The ADC that targets DLL-3 (rovalpituzumab tesirine or Rova-T) is represented in an open-label phase I/II trial [13], which enrolled multiple DLL-3-positive unresectable/advanced solid tumour types refractory to at least one standard treatment. The primary endpoint was to assess the safety and tolerability of Rova-T, an ADC linked to pyrrolobenzodiazepine with a cytotoxic payload that binds to DNA sequences causing lethal lesions, with a dose escalation phase followed by an expansion phase.

The study included 200 participants, 101 of whom had NEC/NET and 21 of whom had NEPC. The remainder, 99, were other solid tumors. Of these, 145 patients received at least one dose of Rova-T with the recommended phase II dose of 0.3 mg/kg every six weeks for two cycles. Sixty-nine were NEC/NET, of whom fourteen were NEPC patients.

Regarding safety data, serious AEs were reported in 52% of patients (n = 59); the most commonly seen were pleural effusions (n=7; 5%), pericardial effusions (n=6; 4%), and dyspnea (n = 5; 3%). Treatment was discontinued due to toxicity in 21% of patients (n=31).

Overall, 21 deaths (14%) were reported. Four of them were directly related to the study drug, including two events of pneumonitis, one of acute respiratory failure, and one of hepatic encephalopathy. Four of them were directly related to the study drug, including two events of pneumonitis, one acute respiratory failure, and one hepatic encephalopathy.

ORR was seen in 10% (n=15) of all treated patients. In a subset analysis of NEC/NET, the ORR was 13% (n=9), with one (7%) partial response seen in NEPC.

The median follow-up in the NEPC group was 4.7 months (range, 0.1-27.1), with a median duration of treatment of three months (2.8-3.1), a median progression-free survival (PFS) of 4.8 months (2.7-5.7), and a median OS of 6.4 months (3.6-9.0). However, after the results of the two phase III trials in advanced small-cell lung cancer, DLL1 ADC development was discontinued due to a lack of survival benefit [22].

Other Targets Currently Under Investigation

Ongoing clinical trials are also investigating other alternative targets. One novel target is B7-H3, a newly identified peripheral membrane protein expressed on activated antigen-presenting cells, known to have a role in T cell response regulation and is believed to be associated with an undesirable prognosis in prostate cancer. An open-label phase I/II clinical trial [14] is currently recruiting patients with advanced solid tumours, including refractory mCRPC, to be treated with MGC018-an anti-B7-H3 ADC linked to duocarmycin as payload, a potent alkylating agent.

Epidermal growth factor receptor (HER2) is also a target of interest in prostate cancer and is currently under investigation in 3 phase I/II clinical trials. The first one is the PETRA trial [17], an ongoing phase I/II, open-label, multi-arm study, investigating poly (ADP-ribose) polymerase inhibitors (PARPi), alone or in combination with anti-HER2 ADCs (trastuzumab-DXd) or anti-TROP2 ADCs (dato-DXd), as well as other anti-cancer agents. It enrolls patients with various advanced solid malignancy types, including PCa, and the primary endpoints are safety, tolerability, and tumour activity of these drugs.

The other clinical trial is the MATCH screening trial [18], a phase II clinical trial in which various types of solid tumours, lymphomas, and myelomas that have progressed following at least one line of standard treatment are genetically tested to identify potential activating and targetable genetic abnormalities. One of the treatment arms is trastuzumab-emtansine, an anti-HER2 ADC linked to maytansinoid-1 and directed to HER2-positive tumours (expressing the HER2 antigen or having an amplified HER2 gene). Safety and anti-tumoural activity are primary endpoints.

Finally, the third ongoing clinical trial is also open-label, phase I/II, the first human trial of A166 [19], an anti-HER2 ADC linked to duostatin-5 (a microtubule inhibitor), in relapsed or refractory HER2-positive tumours. There is a dose escalation and dose expansion phase. Dose-limiting toxicity is one of the primary endpoints of the dose-escalation part.

Discussion

At present, available data on the efficacy of ADCs in prostate cancer favours the use of anti-PSMA ADCs linked to microtubule inhibitors, as demonstrated by an overall PSA decline rate of ≥50% in 14% of patients, as reported by Petrylak et al. [8], and in 8% of the studied population, as reported by Milowsky et al. [9]. Interestingly, Petrylak et al. reported a higher anti-tumor activity and overall survival in the chemotherapy-naïve group of patients, raising the question of whether the use of anti-PSMA ADCs in earlier treatment lines could result in even better outcomes.

However, when looking at the PSMA ADC reporting the best results (PSA decline rate ≥ 50% in 14% of patients in second-line mCRPC), their activity seems, overall, lower than the reported activity of the available standard of care (SoC) options such as second-generation hormonal therapies [23,24] and cytotoxic agents (docetaxel and cabazitaxel) [25,26]. It is important to stress that, to date, there are no head-to-head comparisons between ADCs and SOC treatments in mCRPC, and the nature of cross-trial comparisons is limited.

This overall lower activity of ADCs might be explained by several factors. Some possible explanations include the fact that PSMA ADC does not directly target androgen-mediated pathways, as well as the fact that a rising baseline PSA was not an inclusion criterion for either of the two PSMA ADC trials. Lastly, a narrow therapeutic window may also have contributed to the low responses, with a significant proportion of patients (31%) not receiving the total scheduled PSMA ADC regimen due to AEs, most commonly peripheral neuropathy.

The heterogeneity of the chosen primary endpoints for evaluating ADCs’ activity between the different studies may also have affected the reported results. PSA decline rate was a primary endpoint in PSMA ADC trials, although it is not prospectively validated as a surrogate marker for survival in PCa [27,28].

The overall radiologic response was chosen as an efficacy endpoint for sacituzumab-govitecan, Rova-T, STEAP-1, and TF studies, with some activity reported in the first three cases and none in the last one. In addition, the best endpoint to measure the activity of an ADC in prostate cancer is yet to be defined.

Currently, it is clear that the concept of ADCs as "magical bullets" has not been fully verified in the mCRPC setting, as a wide range of off-target toxicity has been reported. Possible reasons presented by the authors are payload linker instability or the presence of a bystander effect. Further studies are needed to fully understand what aspects should be looked at when choosing an ADC.

## Conclusions

Treatment of mCRPC is challenging, and the response to the majority of the existing treatments remains limited. The use of ADCs may change this reality, although, at present, the available data only favours the use of anti-PSMA ADCs as potentially beneficial. Furthermore, the interpretation and applicability of ADC trials in PCa are limited by a small sample size and study heterogeneity concerning the included population and the primary endpoints used across the different trials. Nevertheless, the results of most prospective ongoing studies are still awaited, and a longer follow-up time is warranted to evaluate the real impact of ADCs in PCa.
